# Comparison of the Unipolar Electrocautery and the Bipolar Sealer in Reducing Blood Loss in Total Knee Arthroplasty: A Prospective, Randomized, Noninferiority Study

**DOI:** 10.1016/j.artd.2024.101509

**Published:** 2024-09-23

**Authors:** Eugene S. Krauss, Ayal Segal, Barry G. Simonson, Nancy Dengler, MaryAnne Cronin

**Affiliations:** a Department of Orthopaedic Surgery, Syosset Hospital, Northwell Health, Syosset, NY, USA; b Zucker School of Medicine, Hofstra/Northwell, Hempstead, NY, USA; c New York Orthopaedic and Spine Center, Great Neck, NY, USA; d Orthopedic Associates of Great Neck, Great Neck, NY, USA

**Keywords:** Bipolar sealer, Unipolar electrocautery, Total knee arthroplasty, Hemostasis

## Abstract

**Background:**

This was a noninferiority trial to evaluate blood loss during total knee arthroplasty (TKA) when using the unipolar electrocautery system compared to the saline coupled bipolar sealer system in primary TKA.

**Methods:**

One hundred sixty-four patients were randomly assigned by a 1:1 ratio to either the unipolar electrocautery system (N = 82) or bipolar sealer system (N = 82). Inclusion criteria included patients scheduled for primary unilateral TKA, preoperative hemoglobin ≥11 mg/dL, preoperative platelet count ≥150,000, age >18 years, and patient willing to complete all study-related procedures. The primary efficacy outcome was estimated blood loss on morning of postoperative day. Secondary efficacy outcomes were comparison between the preoperative hemoglobin and postoperative day 1 hemoglobin, and allogeneic blood transfusions. Additionally, the study collected objective and functional outcomes using the postoperative 2011 Knee Society Score.

**Results:**

The unipolar electrocautery system was not found to be less efficacious than the bipolar sealer system. Mean blood loss for the unipolar electrocautery system was 1062.0 cubic centimeters (cc) (95% confidence limit for the mean: 985.2, 1138.7), and for the bipolar sealer system was 929.4 cc (95% confidence limit for the mean: 841.9, 1016.8). The mean difference in blood loss was 132.6 cc, below the margin of inferiority set at 200 cc. Additionally, there was no difference in patient outcomes as measured by the Knee Society Score.

**Conclusions:**

The safety, efficacy, and outcomes profile of the unipolar electrocautery system compared to the bipolar sealer system were similar. Use of the bipolar sealer system significantly increases surgical cost without any added benefits.

## Introduction

Total knee arthroplasty (TKA) can result in significant blood loss, therefore numerous techniques have evolved to reduce bleeding and promote hemostasis. The unipolar electrocautery system emerged as an adjunct to surgery in 1926 across an entire range of surgical disciplines. The unipolar electrocautery results in the tissue surface temperature exceeding 400°C where the tip touches the tissue, causing generalized disruption of cells and tissue architecture [1].

The use of saline coupled bipolar sealing system represents another approach to reducing blood loss in patients undergoing total joint arthroplasty. This technology uses bipolar radiofrequency energy combined with a continuous-flow saline at the electrode tip to prevent tissue temperatures from exceeding 100°C. The temperature is sufficient to shrink collagen fibers in blood vessel walls, which seals their lumen resulting in hemostasis without surrounding tissue damage [2]. Additionally, this technology has the ability to not only spot coagulate vessels but also broadly paint surfaces that could ooze after the soft tissues have been closed [3].

Arthroplasty has evolved to include techniques not available when the bipolar sealer system was first introduced. The use of intravenous tranexamic acid (IV TXA) during surgery to promote hemostasis is well documented [4,5]. Additionally, the use of neuraxial anesthesia and lowering of transfusion thresholds have become standard of care. Therefore, how does the bipolar sealer perform in conjunction with these other strategies?

The null hypothesis for this study is the difference between the mean estimated blood loss in the unipolar electrocautery and the saline-coupled bipolar sealer is greater than or equal to the margin of non-inferiority of 200 cubic centimeters (cc). Also, we propose that the safety and efficacy profile of the unipolar electrocautery vs the saline-coupled bipolar sealer are clinically equivocal.

The primary objective of this study was to assess whether the unipolar electrocautery system was noninferior to the bipolar sealer system with respect to estimated blood loss. Estimated blood loss was calculated using the gross formula on postoperative day 1 (POD1). Secondary outcomes evaluated were patient objective and functional scores measured by the postoperative Knee Society Score (KSS), delayed wound healing, surgical complications within 90 days postoperatively, the difference between preoperative vs POD1 hemoglobin (Hb), and the need for allogeneic red blood cell transfusions.

## Materials and methods

This institutional review board-approved study was conducted according to US and international standards of Good Clinical Practice (Food and Drug Administration Title 21 part 312 and International Conference on Harmonization guidelines), applicable government regulations and institutional research policies and procedures. The study was registered on ClinicalTrials.gov Protocol Registration System NCT03952546.

### Trial design

This was a prospective, single-center, randomized, single-blinded, noninferiority study to compare the unipolar electrocautery and bipolar sealer system in primary, unilateral TKA. The patient and the postoperative hospital staff were blinded to the treatment arm.

### Study group

**Figure 1:**
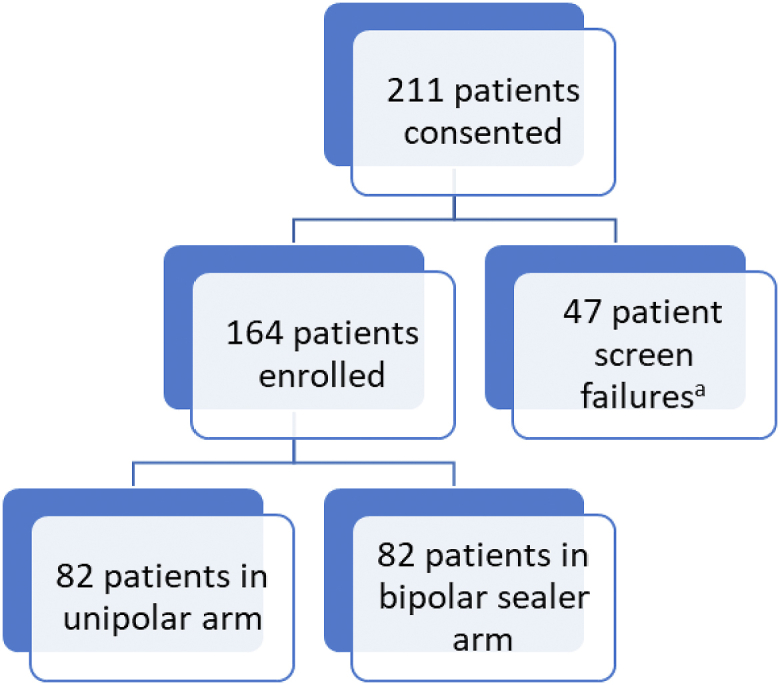
Study enrollment. ^a^Patient not meeting the inclusion criteria or having one or more of the exclusion criteria listed in Table 1.

Between June 2019 and March 2021, 211 patients scheduled for primary TKA signed informed consent to participate in the clinical trial (Fig. 1). Forty-seven patients were excluded from the study as they did not meet the study criteria outlined in Table 1. The Biostatistics Unit developed a randomization procedure using a permuted block design. One hundred sixty-four patients were randomly assigned by a 1:1 ratio to either the unipolar electrocautery system (N = 82) or the bipolar sealer system (N = 82).

**Table 1:** Study inclusion and exclusion criteria.

Inclusion criteria
Patients scheduled for primary unilateral total knee arthroplasty
Preoperative hemoglobin ≥11 mg/dL
Preoperative platelet count of ≥150,000
Age >18
Patient willing to complete all study related procedures
Exclusion criteria
Patients unable to take to aspirin or apixaban for venous thromboembolism prophylaxis for any reason
History of venous thromboembolism (deep vein thrombosis, pulmonary embolism) within 12 months prior to the date of surgery
Mitral valve replacement or aortic valve replacement with additional risk factor for stroke (atrial fibrillation, previous thromboembolism, left ventricular dysfunction, hypercoagulable conditions)
Active cancer
Inherited thrombophilia, eg,: Factor V Leiden, Protein C and S deficiencies, antithrombin deficiency, prothrombin 20210A mutations
Acquired thrombophilia, eg,: lupus anticoagulant, antiphospholipid antibody syndrome
Patients unable to get intravenous tranexamic acid for any reason
Patients requiring anticoagulant treatment prior to surgery
Any medical condition that in the opinion of the investigator would require special fluid management protocols during or after surgery
Patients who are unwilling to undergo blood transfusion, if necessary
Patients unable to have spinal anesthesia, eg,: lumbar fusion, abnormal coagulation test results, stenotic valvular heart disease, severe left ventricular dysfunction or cardiomyopathy, multiple sclerosis, severe scoliosis, ventriculoperitoneal shunting, increased intracranial pressure, allergy to local anesthetics
Patients receiving erythropoietin therapy for anemia
Patients with a contraindication for the pneumatic tourniquet applied in the operating room
Patients who are unable to stop their daily aspirin, aspirin-like products, and/or non-steroidal anti-inflammatory agents 7 days prior to surgery for any reason
Blood transfusion within 1 month of surgery

### Outcomes measured

The primary efficacy outcome was estimated blood loss on the morning of POD1, calculated by the gross formula. POD1 was used for this analysis as TKA surgery is considered a fast-track surgery with our average length of stay being 1.3 days. Laboratory values on POD1 were drawn prior to chemoprophylaxis initiation, as chemoprophylaxis could be a confounding factor in blood loss.

Secondary efficacy outcomes were the comparison between the preoperative Hb and POD1 Hb, and allogeneic blood transfusions. Additionally, the study collected both objective and functional outcomes using the postoperative 2011 KSS. Patients were followed up for 90 days after surgery for adverse events such as venous thromboembolism (VTE), wound infection or delayed wound healing. The study was restricted to 3 surgeons with more than 30 years’ experience to limit variations in the measured outcomes due to differences in surgical technique. The protocol was amended on October 29, 2019, when the third surgeon was added to the study.

### Gross formula measurements

Estimated blood loss was calculated using the gross formula with an estimated total blood volume calculated by the Moore formula. These methods were used to provide a more standardized method of calculating blood loss. The gross formula was calculated using the preoperative hematocrit (Hct_0_) and the POD1 Hct_final_ (Hct_f_) [6]. Estimated blood volume was calculated using the Moore formula. The Moore formula calculates blood volume as a function of body habitus [7]. The equations are as follows:

Moore formula: Estimated blood volume = Weight in kilograms x body habitus

Body habitus: Men: obese = 55; thin = 65; normal = 70; muscular = 75; women: obese = 55; thin = 60; normal = 65; muscular = 70

Gross formula: Estimated blood volume x [(Hct_0_-Hct_f_)/Hct_av_] Hct_av_ = the average of the preoperative and POD1 Hct.

### Study conduct

Current preoperative, intraoperative, and postoperative blood management strategies were consistent for all subjects. Preoperatively patients taking anticoagulants or antiplatelets, except aspirin (ASA) 81 mg daily, were excluded from the study as these medications could adversely affect intraoperative and postoperative bleeding. Patients were instructed to discontinue ASA, medications containing ASA-like products, and nonsteroidal anti-inflammatory drugs 7 days prior to surgery. Patients who were unable to stop these products for any reason were excluded from the study. Intraoperative blood management strategies were consistent for all patients to avoid any confounding factors which could affect postoperative transfusion requirements.

Intraoperatively, anesthetic, pharmacologic, and surgical techniques were employed to minimize blood loss. Neuraxial anesthesia has been shown to reduce intraoperative blood loss compared to general anesthesia [8]. Any patients with preoperative contraindications to neuraxial anesthesia were excluded from the study. Unfortunately, due to technical issues in the operating room, some patients were unable to receive neuraxial anesthesia and were given general anesthesia. Though a rare occurrence, these patients remained on study and were included in the study results as this was an intent-to-treat study design.

Pharmacologic techniques to minimize blood loss included the administration of IV TXA intraoperatively. The first dose of 1000 mg/50cc Normal saline was given prior to incision and the second dose was given when the pneumatic tourniquet was released or at least 60 minutes after the first dose [9]. Patients not meeting departmental criteria for IV TXA (Table 2) were excluded from the study.

**Table 2:** Exclusion criteria for intravenous tranexamic acid.

Active cancer
Allergy to tranexamic acid
OR any 2 or more of the following:
History of thromboembolism within prior 12 months
Mitral valve replacement or aortic valve replacement including transcatheter aortic valve replacement (TAVR) plus additional risk factors for stroke (atrial fibrillation, previous thromboembolism, left ventricular dysfunction, hypercoagulable conditions)
Inherited thrombophilia, eg,: Factor V Leiden, Protein C deficiency, Protein S deficiency, Antithrombin deficiency, prothrombin 20210A mutation
Acquired thrombophilia, eg,: lupus anticoagulant, antiphospholipid antibody syndrome, Human immunodeficiency virus
History of myocardial infarction, cardiac stents or coronary artery bypass surgery
History of stroke or transient ischemic attack

With respect to surgical techniques used to minimize blood loss, the pneumatic tourniquet was applied prior to incision and inflated to 250 millimeters mercury (mmHg) to maintain a bloodless field. Upon release of the tourniquet bleeding vessels were sealed with either the bipolar sealer system or the unipolar electrocautery system as determined by subject randomization.

As per institutional guidelines all patients received 2 postoperative normal saline 500-mL boluses, the first in postanesthesia care unit and the second 4 hours later, as well as Lactated Ringers IV at 100 mL/h until POD1. The allogeneic blood transfusion policy was consistent for all patients. Patients were transfused when the Hb was less than or equal to 7 g/dL. For Hb greater than 7 g/dL to less than 8 g/dL patients were treated only if they are exhibiting clinical symptoms related to the anemia or if there is a rapid decline in Hb.

The KSS objective and functional measurements were collected at the 8-week postoperative visits, or the office visit closest to this timepoint. Patients were followed for 90 days from the date of surgery for any device-related adverse events, surgical site infections, delayed wound healing or VTE events.

VTE prophylaxis consisted of both chemical and mechanical measures. All patients had an intermittent pneumatic compression device applied in the operating room to the nonoperative extremity. At the end of the surgical case, after the dressing had been applied, the intermittent pneumatic compression was applied to the operative extremity. intermittent pneumatic compression devices were used while patients were in bed.

All patients were started on VTE prophylaxis POD1. POD1 laboratory results were used for the study calculations as these were drawn prior to initiation of VTE prophylaxis. Low-risk patients were prescribed ASA 81 mg twice a day (BID) for 6 weeks. High-risk patients were prescribed apixaban 2.5 mg BID for 2 weeks followed by ASA 81 mg BID for 4 weeks [10].

### Statistical analysis

Analysis was conducted using SAS, version 9.4 (SAS Institute, Inc, Cary, North Carolina).

Summary statistics for the study sample are presented as median, lower quartile, and upper quartile for measured variables and frequencies with percentages for categorical variables.

The sample size for this study was 164 subjects (n = 82 per group). As a noninferiority trial, the unipolar electrocautery system was considered noninferior to bipolar sealer system if the difference in the mean estimated blood loss as calculated by gross formula was less than 200 cc (δ = margin of inferiority = 200 cc). The test for noninferiority was carried out using a 2-tailed 95% confidence interval for the difference (δ = Unipolar electrocautery system – bipolar sealer system) in the estimated blood loss between the 2 groups. δ is the margin of inferiority and will be set at 200 cc. This test for noninferiority was only performed for the primary study endpoint of estimated blood loss; all other secondary variables were tests of superiority of unipolar electrocautery system vs bipolar sealer system. Results were considered statistically significant at the *P* < .05 level of significance. The primary analysis was based on the intention-to-treat population. The intention-to-treat population was defined as any subject who was randomized.

## Results

There were no significant demographic differences between the 2 groups. Descriptive data for each group are shown on Table 3. A total of 14 patients received general anesthesia, 6 patients in the bipolar sealer arm and 8 patients in the unipolar electrocautery arm.

**Table 3:** Baseline demographics

Criteria	Unipolar electrocautery system	Saline coupled bipolar sealer
N	82	82
Women	45	54
Men	37	28
BMI 18.5-29.9	34	29
BMI ≥30	48	52
BMI <18.5	0	1
Spinal anesthesia	74	76
General anesthesia	8	6

	MEAN	STD DEV	MEAN	STD DEV
Age	66.1	8.9	64.3	8.3
BMI	31.6	5.3	32.4	7.4
Preop Hb	13.9	1.2	13.9	1.1
Preop Hct	42.5	3.4	42.6	3.3
Preop Plt	248.3	52.7	249.0	49.3

N, number; STD DEV, standard deviation are presented as mean + standard deviation; BMI, body mass index; Preop Hb, preoperative hemoglobin; Preop Hct, preoperative hematocrit; Preop Plt, preoperative platelets (K/uL).

### Primary outcome

The unipolar electrocautery system was not found to be less efficacious than the bipolar system. Mean blood loss for the unipolar system was 1062.0 cc (95% confidence limit for the mean: 985.2, 1138.7), and for the bipolar sealer system, it was 929.4 cc (95% confidence limit for the mean: 841.9, 1016.8) This result did not reach statistical significance (*P* = .1254). The mean difference in blood loss was 132.6 cc, below the margin of inferiority set at 200 cc.

### Secondary outcomes

Only one subject treated with the unipolar electrocautery system required an allogeneic red blood cell transfusion. No subjects treated with the bipolar device required a transfusion. There was no difference in estimated blood loss as measured by Hb.

There was no difference in wound infections. All patients who developed cellulitis were treated with one course of antibiotics with complete resolution. There were no superficial or deep wound infections in either treatment group. Additionally, there were no device-related adverse events. One patient in the bipolar sealer arm was diagnosed with a distal VTE (Table 4).

**Table 4:** Secondary outcomes.

Criterion	Unipolar electrocautery	Bipolar sealer	*P* value
Mean decrease Hb (CLM)	1.3878 (1.2164, 1.5592)	1.1617 (0.9743, 1.3491)	.0782
Mean total KSS (CLM)	61.7945 (58.1966, 65.3925)	56.4583 (51.5726, 61.3441)	.0819
Cellulitis within 90 d	3.7% (3/82)	1.2% (1/82)	.6204

Hb, hemoglobin (g/dL); CLM, 95% confidence limit for the mean; KSS, knee society score.

## Discussion

The results of our study concluded that there were no clinically significant differences in blood loss and patient reported outcomes between the unipolar electrocautery system and bipolar sealer system. The safety profile of the 2 devices was also similar. Patients were contacted 90 days after surgery to assess for any adverse events. All patients were able to be contacted except for 3; however, these patients had been seen at the 8-week postoperative visit. One patient in the bipolar sealer arm developed a wound hematoma on POD9. The patient had been taking apixaban for VTE prophylaxis. The apixaban was discontinued and the patient was started on ASA 81 mg BID for the remainder of the treatment period. The hematoma resolved without any further intervention. One patient in the bipolar sealer arm was diagnosed with a distal DVT on POD74. The patient was treated with anticoagulation without further sequelae. None of these events were considered related to the study devices.

The bipolar sealer device adds an additional cost of $453 to each TKA case. However, our study showed this additional cost did not result in any added benefits to patient outcomes. The elimination of the bipolar sealer device results in a significant cost reduction for the hospital, with annual cost savings in excess of $400,000. For the entire health system, these cost savings would exceed $2,000,000.00.

When first introduced the bipolar sealer device was promoted as providing enhanced hemostasis while reducing tissue damage. The advantages claimed to be diminished thermal injuries, reduced charring, less tissue necrosis, reduced time of operation, ease of visualization of vessels being sealed, no systemic morbidity risks, and the absence of foreign material left at the surgical site [11]. There have also been anecdotal references that the use of the bipolar sealer allowed for a decrease in postoperative pain and swelling, leading to quicker rehabilitation, and improved overall patient outcomes following TKA. Rosenberg [12] found that the bipolar sealer technology not only decreased blood loss and associated transfusion requirements, but, perhaps more significantly, reduced postoperative pain and swelling. This was characterized by reduced incidence of hemarthrosis and more rapid rehabilitation. However, our study found there were no clinically significant differences in postoperative objective or functional clinical outcomes as measured by KSS.

There have been randomized clinical trials which have challenged the efficacy of the bipolar sealer system and do not support superiority of this method when compared to standard unipolar electrocautery system. In 2016, Seviciu et al [13] published the results of a clinical trial evaluating the use of the bipolar sealer in combination with IV TXA. Standard electrocautery was used to control bleeding in the operating room for all study subjects, thereby eliminating a head-to-head comparison of these 2 cautery methods.

The conflicting results of these clinical trials are multifactorial, highlighting the rapidly changing landscape of orthopaedic surgery. Except for Seviciu’s clinical trial, many of these studies predate the use of IV TXA in orthopaedic surgery and its dramatic effect on hemostasis. IV TXA has been shown in multiple prospective and retrospective studies to decrease the incidence of bleeding and the need for allogeneic blood transfusions after total hip and knee arthroplasty [9]. The use of IV TXA in TKA patients has been the standard of care practice since March 2013 at our institution. Current trends in allogeneic blood transfusion protocols have also changed. Patients are now transfused at lower hemoglobin levels. Additionally, safety data are lacking in these publications as most patients were observed in the hospital setting only. Long-term follow-up on clinical outcomes such as range of motion and functional assessment scores were not reported in many of the trials.

### Limitations

A basic weakness of noninferiority trials is that poor conduct of the trial or deviations from the protocol could result in false rejection of the null hypothesis that the experimental treatment is inferior. This weakness was mitigated by strict adherence to the protocol to prevent any protocol deviations and constant monitoring of all protocol activities. Finally, the stringent inclusion and exclusion criteria limited the study population to patients with fewer comorbidities. Therefore, these findings cannot be extrapolated to all arthroplasty patients. To our knowledge, this is the only study to assess perioperative hemostasis, the safety profile for 90 days postoperatively, and patient objective and functional outcomes.

## Conclusions

The safety, efficacy and outcomes profile of the unipolar electrocautery system compared to the bipolar sealer system were similar. The use of the bipolar sealer system added to the cost of surgery without any added benefit in surgical outcomes.

## Acknowledgments

The authors wish to thank their colleagues Debra Schulman, RN, Marie Marzano, RN, and Renne Rodriquez, NP for their support and assistance.

## Funding

This study was self-funded by the Department of Orthopedic Surgery at Syosset Hospital, 10.13039/100019311Northwell Health.

## Conflicts of interest

Krauss and Segal report receiving royalties from and being pain consultants for Enovis. All other authors declare no potential conflicts of interest For full disclosure statements refer to https://doi.org/10.1016/j.artd.2024.101509.

## CRediT authorship contribution statement

**Eugene S. Krauss:** Writing – review & editing, Supervision, Project administration, Methodology, Investigation, Data curation, Conceptualization. **Ayal Segal:** Writing – review & editing, Supervision, Project administration, Methodology, Investigation, Conceptualization. **Barry G. Simonson:** Writing – review & editing, Supervision, Project administration, Methodology, Investigation, Data curation. **Nancy Dengler:** Writing – review & editing, Writing – original draft, Supervision, Project administration, Methodology, Investigation, Data curation, Conceptualization. **MaryAnne Cronin:** Writing – review & editing, Writing – original draft, Supervision, Project administration.

## Appendix A Supplementary data

Conflict of Interest Statement for Cronin

Conflict of Interest Statement for Dengler

Conflict of Interest Statement for Krauss

Conflict of Interest Statement for Segal

Conflict of Interest Statement for Simonson
